# Polygenic risk scores for premagnetic resonance imaging risk stratification in men with clinically suspected prostate cancer

**DOI:** 10.1093/jnci/djag027

**Published:** 2026-02-02

**Authors:** Max P Fischer, Alena Mayer, Alice Braun, Julia Kraft, Carla M Hansen, Georg Lukas Baumgärtner, Patrick Asbach, Anwar R Padhani, Tobias Penzkofer, Frank König, Swapnil Awasthi, Marcus R Makowski, Stephan Ripke, Charlie Alexander Hamm

**Affiliations:** Department of Psychiatry, Laboratory for Statistical Genetics, Charité – Universitätsmedizin Berlin, Corporate Member of Freie Universität Berlin and Humboldt-Universität zu Berlin, Berlin, Germany; Department of Psychiatry, Laboratory for Statistical Genetics, Charité – Universitätsmedizin Berlin, Corporate Member of Freie Universität Berlin and Humboldt-Universität zu Berlin, Berlin, Germany; Department of Psychiatry, Laboratory for Statistical Genetics, Charité – Universitätsmedizin Berlin, Corporate Member of Freie Universität Berlin and Humboldt-Universität zu Berlin, Berlin, Germany; Department of Psychiatry, Laboratory for Statistical Genetics, Charité – Universitätsmedizin Berlin, Corporate Member of Freie Universität Berlin and Humboldt-Universität zu Berlin, Berlin, Germany; Department of Internal Medicine, Clinic for Nephrology and Intensive Care, Charité – Universitätsmedizin Berlin, Corporate Member of Freie Universität Berlin and Humboldt-Universität zu Berlin, Berlin, Germany; Department of Radiology, Charité – Universitätsmedizin Berlin, Corporate Member of Freie Universität Berlin and Humboldt-Universität zu Berlin, Berlin, Germany; Department of Radiology, Charité – Universitätsmedizin Berlin, Corporate Member of Freie Universität Berlin and Humboldt-Universität zu Berlin, Berlin, Germany; Paul Strickland Scanner Centre, Mount Vernon Cancer Centre, Northwood, United Kingdom; Department of Radiology, Charité – Universitätsmedizin Berlin, Corporate Member of Freie Universität Berlin and Humboldt-Universität zu Berlin, Berlin, Germany; Department of Urology, Uro-Oncology, Robot-Assisted and Focal Therapy, University Clinic Magdeburg, Magdeburg, Germany; Department of Psychiatry, Laboratory for Statistical Genetics, Charité – Universitätsmedizin Berlin, Corporate Member of Freie Universität Berlin and Humboldt-Universität zu Berlin, Berlin, Germany; Department of Diagnostic and Interventional Radiology, Faculty of Medicine, Technical University of Munich, Munich, Germany; Department of Psychiatry, Laboratory for Statistical Genetics, Charité – Universitätsmedizin Berlin, Corporate Member of Freie Universität Berlin and Humboldt-Universität zu Berlin, Berlin, Germany; Berlin Institute of Health, Berlin, Germany; Department of Radiology, Charité – Universitätsmedizin Berlin, Corporate Member of Freie Universität Berlin and Humboldt-Universität zu Berlin, Berlin, Germany; Berlin Institute of Health, Berlin, Germany

## Abstract

**Background:**

Men with suspected prostate cancer undergo magnetic resonance imaging (MRI) before biopsy. However, approximately 30%-50% of MRIs are negative (Prostate Imaging–Reporting and Data System [PI-RADS] score 1-2), representing a challenge for MRI resource utilization. This study evaluates prostate cancer polygenic risk scores and clinical markers to optimize MRI utilization.

**Methods:**

In this prospective study, 500 cancer-suspected men of Western European descent scheduled for MRI (September 2017-December 2022) were enrolled. Exclusions included prior prostate cancer diagnosis, missing serum prostate-specific antigen (PSA), or PSA levels of at least 25 ng/mL. Patient-specific prostate cancer polygenic risk scores were calculated using genotype data obtained from saliva-derived DNA samples. Participants were grouped as MRI negative and positive (PI-RADS score 3-5). Logistic regression was used to calculate odds ratios (ORs) and to build multivariable risk models, including age, PSA, and polygenic risk scores for MRI positivity. Clinical utility was tested in a holdout test set using decision curve analysis.

**Results:**

A total of 386 men (median age = 65 years, interquartile range [IQR] = 53-77 years) were eligible for analysis, which showed statistically significant associations between prostate cancer polygenic risk scores (OR = 1.56, 95% confidence interval [CI] = 1.23 to 1.98; *P* < .001) with MRI positivity, while PSA alone did not (OR = 1.17, 95% CI = 0.93 to 1.46; *P* = .18). The highest net benefit was shown using a multivariable age and prostate cancer polygenic risk score model, increasing the proportion of MRI-positive men by 14% compared with PSA alone (60% and 46%, respectively; *P* = .011).

**Conclusions:**

Genotype-informed risk stratification using prostate cancer polygenic risk scores could increase the proportion of cancer-suspicious findings at MRI, while identifying those who could safely avoid unnecessary MRI.

## Introduction

Prostate cancer is the most common cancer and the fifth leading cause of cancer-related deaths among men in Europe.^1^ With early detection, prostate cancer has excellent survival rates. Current prostate cancer early detection guidelines recommend prostate-specific antigen (PSA) testing.^2^ In men with elevated serum PSA levels, the current diagnostic standard is to perform prebiopsy multiparametric magnetic resonance imaging (MRI), enabling MRI-directed biopsies for the detection of clinically significant prostate cancer while avoiding unnecessary biopsies in a large proportion.^3^ However, up to 50% of men with suspected cancer because of PSA elevations are MRI-negative (Prostate Imaging–Reporting and Data System [PI-RADS] score 1-2), with subsequent biopsies showing a low prevalence of grade group of at least 2 prostate cancers.^4^ These men have undergone unnecessary investigations with negative consequences for the individuals concerned and their health systems. As MRI demand increases, there is a need to select men to enable appropriate utilization of health-care resources.^5^

Genetic factors play a substantial role in prostate cancer etiology.^6^ Prostate cancer is recognized as a genetically complex disease with multiple rare and common genetic variations.^7^ To date, many rare mutations with a prevalence of less than 5% have been identified, including mismatch repair and homologous recombination genes.^7^ The impact of common mutations on prostate cancer development has been studied in genome-wide association studies (GWAS). Overall, GWAS have revealed 269 single-nucleotide polymorphisms (SNPs) associated with prostate cancer, which enable patient-specific polygenic risk score calculations.^8^ In a recent study, 97.5% of prostate cancer deaths by age 75 years had a positive family history or were in the top 2 quartiles of polygenic risk scores, and the clinical validity of the polygenic risk scores has been suggested in recent meta-analyses.^9^^,^^10^ However, its clinical utility as a tool for pre-imaging risk stratification in the context of the diagnostic MRI pathway is not well studied.^11^

Therefore, we aimed to associate prostate cancer polygenic risk scores alone and in combination with clinical parameters with MRI outcomes in cancer-suspected men scheduled for prostate MRI.

## Methods

This was a prospective, observational study conducted at a tertiary academic center between September 2017 and December 2022. Our institution (EA1/232/19) granted ethical approval, and informed consent was acquired upon enrollment. Study data collection and workflow were performed in a predetermined procedure (Figure S1 and Study data management section in Supplemental file). A preemptive sample size calculation aimed for 60%-70% power to detect genetic variants with medium effects (odds ratio [OR] > 1.21) at genome-wide significance.

### Participants

Consecutive men aged 18 years and older scheduled for MRI were recruited either at the referring urology or radiology departments. Participants were eligible for enrollment if they had been referred with a clinical suspicion of prostate cancer based on an elevated serum PSA level, an abnormal digital rectal prostate examination, and/or transrectal ultrasound. The study was limited to men of Western European descent, as the most recent prostate cancer GWAS at trial initiation was conducted in a Western European population, and given our study’s exploratory nature, we focused on a single population to better examine the polygenic risk scores and PI-RADS correlation without introducing potential population-based bias.^12^ Recruitment was paused from December 2020 until April 2022 because of COVID-19 regulations. Men with poor quality SNP data, known prostate cancer, serum PSA level of at least 25 ng/mL, or missing PSA values were excluded.

### Multiparametric MRI

Imaging was performed on 3 Tesla MRI scanners (Magnetom Skyra, Siemens Healthineers) using T2-weighted, diffusion-weighted, and dynamic contrast-enhanced sequences. Specifically, axial and coronal T2-weighted images (3.0 × 0.47 × 0.47 mm) and axial diffusion-weighted images at b-values of 0, 50, 500, 1000 s/mm^2^ with mono-exponentially calculated apparent diffusion coefficient maps, and extrapolated high b-value images (3.0 × 1.4 × 1.4 mm, 22 cm FoV, calculated b = 1400 s/mm^2^) were used. MRIs were reported in consensus by 2 radiologists (5 and ≥15 years of experience), using PI-RADS v2.0 or 2.1 depending on the examination date. Men were then stratified into MRI-negative (PI-RADS score 1-2) and MRI-positive (PI-RADS score 3-5) groups.^2^ Prostate volume and PSA density were assessed using segmentations at MRI. Overall, the MRI quality was of sufficient diagnostic quality (prostate imaging quality score ≥ 3^13^) in 96% (370 of 386) or good/optimal diagnostic quality (prostate imaging quality ≥ 4) in 65% (251 of 386).

### Genetic testing

#### Sampling, genotyping, and quality control

Saliva samples were collected using OraGene OG-510 and OG-610 kits (Genotek), and DNA was extracted by LGC Genomics, Germany. DNA preparation included quantification and quality assessment using the PicoGreen dsDNA-Assay (Thermo Fisher Scientific) and NanoDrop Microvolume Spectrophotometers (Thermo Fisher Scientific), respectively. DNA integrity was evaluated in randomly selected participants via agarose gel electrophoresis.

Genome-wide genotyping and quality control were performed according to current standards, with detailed methods provided in the Supplementary methods (Genetic Sampling and Sample Management subsection). A principal component analysis via EIGENSTRAT^14^ was conducted to assess population stratification, resulting in the exclusion of 13 non-European participants. We imputed missing SNPs with the European Haplotype Reference Consortium panel (1.1),^15^ in EAGLE (2.4.1),^16^ and MINIMAC3.^17^ An additional principal component analysis was performed postimputation to derive the first 4 principal components to control for remaining population stratification in the developed prediction models.

#### Polygenic risk score calculation

For polygenic risk score calculation, the PLINK toolset and metadata from the latest prostate cancer GWAS meta-analysis were used.^8^^,^^18^ GWAS derived effect sizes for all SNPs with a minor allele frequency of more than 5% were recalculated using the continuous shrinkage approach, with default settings and the European 1000 linkage disequilibrium reference matrix.^19^ The constructed prostate cancer polygenic risk score contains 269 SNPs. Additionally, we replicated the polygenic risk score used in the BARCODE1 study.^20^^,^^21^

### Prediction model development

All predictor variables were *z* transformed and used for model training. The data were split into a training set and a holdout test set consisting of 80% (*n* = 309) and the last 20% (*n* = 77) of consecutive participants, respectively.

Following logistic regression testing, 2 nongenetic prediction models (M) were developed, using age as an independent variable alone (M1) and in combination with serum PSA (M2). In addition, both models had genetic versions denoted as M1g and M2g, respectively, where PRS and the first 4 principal components were added as independent variables (Table S1). Two additional models were developed by adding PSA density to M1 (M3) and by further adding genetic variables (M3g) (Supplementary materials). Fivefold cross-validation was used for model training and validation, whereas the holdout test set was used for assessing clinical utility.

### Statistical analysis

The primary outcome of the study was the association of prostate cancer polygenic risk score, alone and with clinical parameters, with PI-RADS scores 3-5 (positive) and 1-2 (negative) at MRI. Secondarily, the study assessed the association between the developed models and PI-RADS scores 1-3 and 4-5.

First, odd ratios (ORs) and corresponding 95% confidence intervals (CIs) were calculated in the training set to assess the likelihood of positive MRI findings for each independent variable. Nagelkerke pseudo-R^2^ (NKR^2^) was computed to gauge the explanatory power of polygenic risk scores in MRI-based prostate cancer risk variability. Second, individual prediction models were evaluated in the holdout test set using bootstrapping (2000 repetitions) to compute area under the receiver operating characteristic curve (AUC), sensitivity, specificity, intercept, and slope. Additionally, AUC and NKR^2^ were calculated using the polygenic risk scores used in the BARCODE1 study.^21^ Third, risk stratification was conducted by training polygenic risk score–based models and clustering into risk quintiles. Odd ratios and 95% confidence intervals for MRI-positivity were estimated for each quintile, with the average risk quintile (41%-60% polygenic risk score quintile) chosen as the reference group. Polygenic risk score quintiles were adjusted for age and the first 4 principal components. Lastly, the clinical utility of individual prediction models was assessed using decision curve analysis and by comparing proportions of MRI findings among different decision strategies through bootstrapping (2000 repetitions).

Continuous variables were presented as median and interquartile range. Statistical significance was determined using the Wilcoxon, Fisher exact and DeLong tests, with 1-sided *P* values less than .05 considered statistically significant. All analyses were performed using R v4.2.2.

## Results

### Sample characteristics

**Figure 1.**
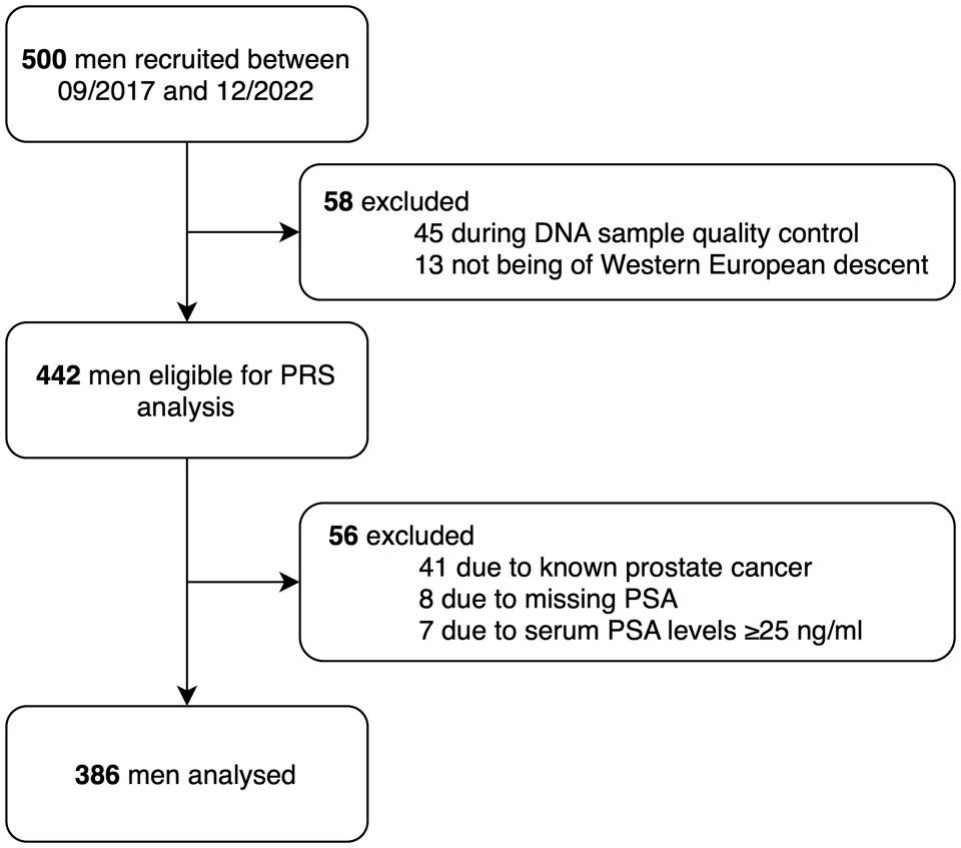
Study profile. Abbreviation: PSA = serum prostate-specific antigen.

A total of 500 men were enrolled in the study of which 114 had to be excluded as detailed in Figure 1. The final cohort consisted of 386 men, 46% (179 of 386) of whom had a positive MRI, a median age of 65 years (interquartile range [IQR] = 60-72 years), and a median serum PSA level of 6.1 ng/mL (IQR = 4.79-8.18 ng/mL; Table 1 and Table S2). Sample characteristics for the training and test sets are reported in Table S3.

**Table 1.** Sample characteristics

	PI-RADS score 1-2 vs 3-5	
Characteristics	MRI-negative men (*n* = 207)	MRI-positive men (*n* = 179)
Age, median (IQR), y	65 (60-71)	67 (60-73)
Body mass index, median (IQR), kg/m²	25.8 (24.5-28.4)	25.9 (23.9-29.4)
Missing	6	10
First-degree *prostate cancer* family history, No. (%)		
No	66 (32)	58 (32)
Yes	21 (10)	20 (11)
Missing	120	101
First-degree breast or ovarian cancer family history, No. (%)		
No	74 (36)	74 (41)
Yes	15 (7)	9 (5)
Missing	118	96
Previous biopsy, No. (%)		
No previous biopsy	140 (72)	154 (89)
Previous biopsy and no malignancy	54 (28)	20 (11)
Missing	13	5
Intake of 5 α-reductase-inhibitors, No. (%)	17 (8)	6 (3)
Missing	1	0
Serum prostate-specific antigen, median (IQR), ng/mL	6.1 (4.7-8.0)	6.1 (4.9-8.5)
Prostate volume, median (IQR), ml	60 (46-74)	40 (32-55)
Prostate-specific antigen density, median (IQR), ng/mL/cc	0.10 (0.07-0.13)	0.15 (0.10-0.21)
Scaled polygenic risk scores, median (IQR)	−0.23 (−0.89 to 0.40)	0.22 (−0.38 to 0.94)
PI-RADS version, No. (%)		
2	82 (40)	80 (45)
2.1	125 (60)	99 (55)
PI-RADS score, No. (%)		
1	6 (3)	0
2	201 (97)	0
3	0	44 (25)
4	0	79 (44)
5	0	56 (31)

Abbreviations: IQR = interquartile range; PI-RADS = Prostate Imaging–Reporting and Data System.

### Association of individual demographic, clinical, and genetic markers with MRI findings

**Figure 2.**
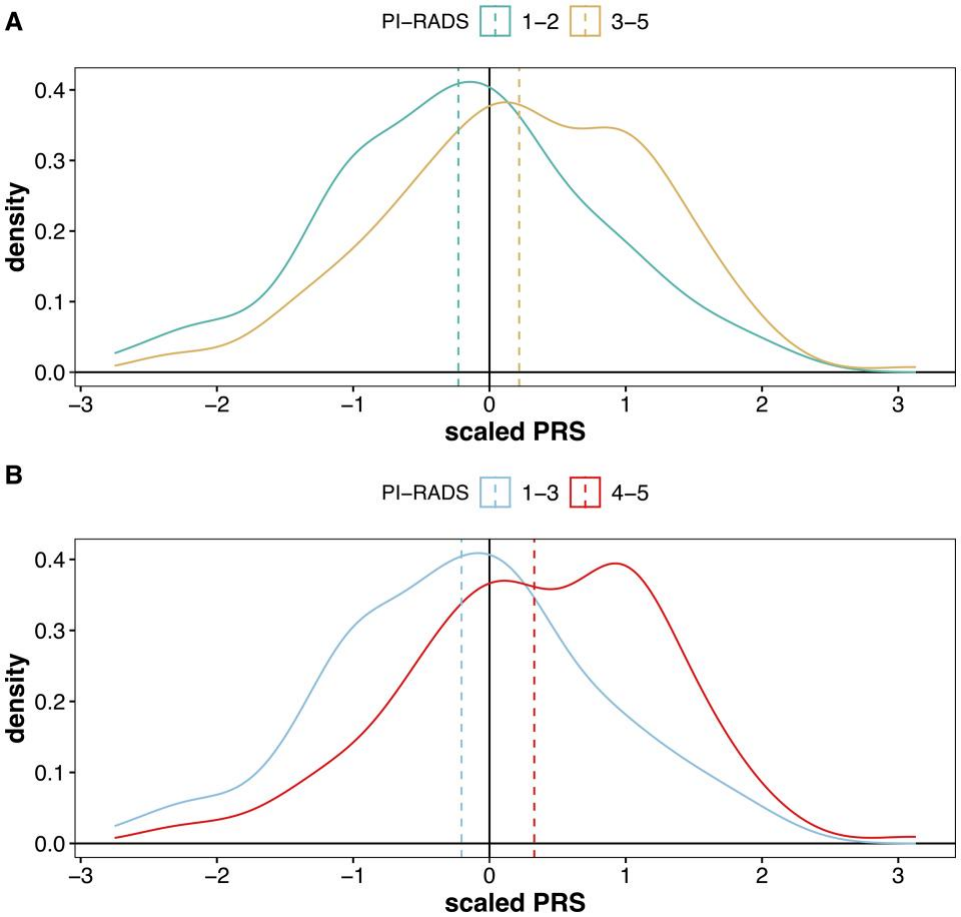
Relationship between prostate cancer–associated polygenic risk scores and magnetic resonance imaging findings. PRS density curves separated by **(A)** negative (PI-RADS score 1-2) and positive (PI-RADS score 3-5) and **(B)** PI-RADS score 1-3 and PI-RADS score 4-5 MRI findings. The dotted vertical line represents the median prostate cancer-PRS for each curve. Note how the two PI-RADS groups statistically significantly differ in terms of polygenic risk for prostate cancer (*P* < 0.001). A breakdown of the individual PI-RADS scores is visualized in Figure S2. Abbreviations: PI-RADS = Prostate Imaging–Reporting and Data System; PRS = polygenic risk score.

**Figure 3.**
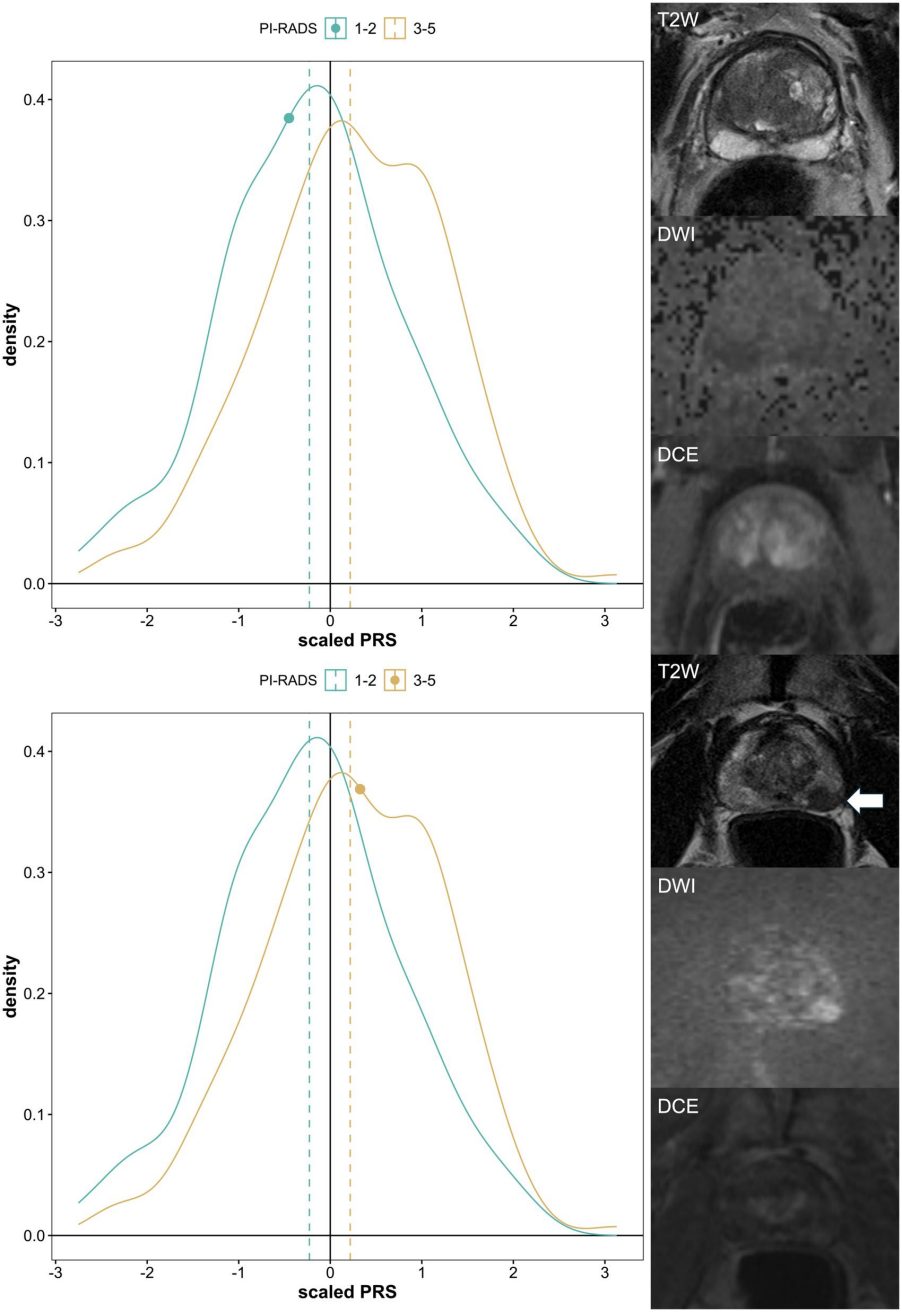
Case examples of men with low and intermediate or high risk for prostate cancer using polygenic risk scoring. Shown are men with PRS indicating low **(A)** and intermediate/high **(B)** risk for prostate cancer (dot on PRS density curve, respectively). Case A is a man aged 75 years with a serum PSA level of 5.46 ng/mL and a PSA density of 0.17 presenting a scaled PRS of −0.451 and a negative magnetic resonance imaging scan (PI-RADS score 2). Case B is a man aged 59 years with a serum PSA level of 2.84 ng/mL and a PSA density of 0.02 presenting a scaled PRS of 0.326 and a 1.3 mm PI-RADS 4 lesion in the left peripheral zone at MRI (arrow). MRI was recommended because of positive family history. Abbreviations: DCE = dynamic-contrast enhanced images; DWI = diffusion-weighted images (calculated b = 1400 s/mm^2^); PI-RADS = Prostate Imaging–Reporting and Data System; PRS = polygenic risk score; PSA = prostate-specific antigen; T2W = T2-weighted images.

On a group level, the prostate cancer polygenic risk score distinguished between MRI-negative and -positive men with an odds ratio of 1.56 (95% CI = 1.23 to 1.98; *P* < .001) (Figure 2); our case examples show a similar trend (Figure 3). Findings for PI-RADS scores 1-3 vs 4-5 were similar with an odds ratio of 1.54 (95% CI = 1.2 to 1.97; *P* < .001) (see Figure S2 for difference of individual PI-RADS). Notably, PSA levels between MRI-negative and -positive men were similar with an odds ratio of 1.17 (95% CI = 0.93 to 1.46; *P* = .18), while an improved odds ratio of 1.29 (95% CI = 1.03 to 1.63; *P* = .03) was shown for men with PI-RADS scores 1-3 vs 4-5 (Table S4).

**Figure 4.**
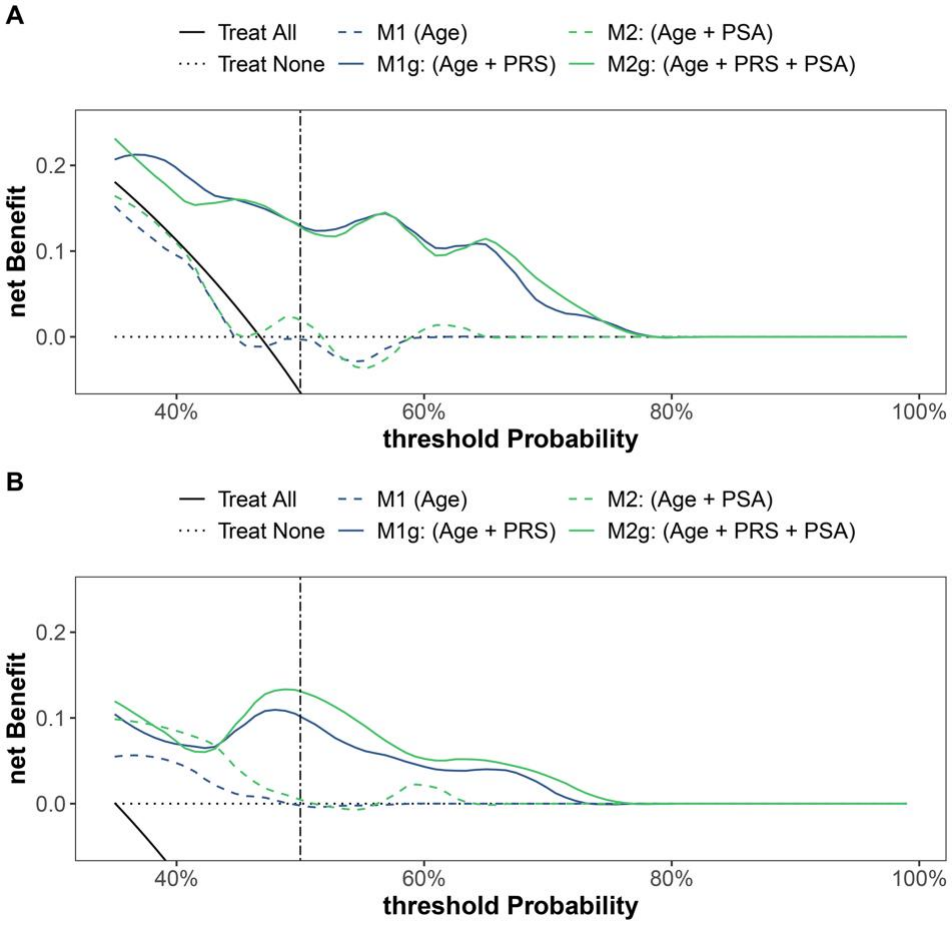
Decision curve analysis comparing clinical utility of different decision strategies for pre-MRI decision-making men with suspected prostate cancer. Decision curve analysis was performed in the holdout test set of 77 men **(A)** for the outcome of PI-RADS scores 1-2 vs 3-5 and **(B)** for PI-RADS scores 1-3 vs 4-5. Decision curve analysis is used to evaluate prediction models by comparing their net benefit. It simulates 2 scenarios: in one all the men would undergo imaging (treat all), and in the other, none would (treat none). Clinically useful decision strategies lie above these scenarios. The graph gives the expected net benefit per participant relative to image none. At a 50% threshold for MRI (every other MRI scan shows PI-RADS score 3-5 lesions), all genetic models had a higher net benefit compared with their nongenetic counterparts. Abbreviations: MRI = magnetic resonance imaging; PI-RADS = Prostate Imaging–Reporting and Data System; PSA = prostate-specific antigen; PRS = polygenic risk score.

When incorporated into prediction models, the polygenic risk score was a statistically significant predictor in all models, whereas age only predicted MRI risk when combined with polygenic risk scores (M1g-M2g; Table 2 and Figure 4). In contrast, when using a PI-RADS cutoff of 1-3 vs 4-5, age also became a statistically significant predictor and the predictive power of all models increased (Table 2). PSA density was a significant predictor in all models, as detailed in Table S5.

**Table 2.** Association of independent markers with positive magnetic resonance imaging findings^a^

	PI-RADS score 1-2 vs. 3-5				PI-RADS score 1-3 vs. 4-5			
	Non-genetic models		Genetic models		Non-genetic models		Genetic models	
	M1	M2	M1g	M2g	M1	M2	M1g	M2g
Marker	OR (95% CI)	OR (95% CI)	OR (95% CI)	OR (95% CI)	OR (95% CI)	OR (95% CI)	OR (95% CI)	OR (95% CI)
Age	1.24 (0.99 to 1.56)	1.21 (0.96 to 1.53)	1.30* (1.02 to 1.65)	1.28* (1.00 to 1.63)	1.45** (1.14 to 1.86)	1.40** (1.09 to 1.80)	1.52** (1.18 to 1.98)	1.47** (1.14 to 1.92)
Serum prostate-specific antigen		1.12 (0.89 to 1.42)		1.10 (0.86 to 1.40)		1.22 (0.96 to 1.55)		1.23 (0.96 to 1.59)
Polygenic risk score for prostate cancer			1.58*** (1.24 to 2.04)	1.58*** (1.24 to 2.03)			1.59*** (1.23 to 2.07)	1.59*** (1.23 to 2.08)

Abbreviations: CI = confidence interval; OR = odds ratio; PI-RADS = Prostate Imaging–Reporting and Data System.

a Nongenetic models used age alone (M1) and in combination with serum PSA (M2). Both models had genetic versions denoted as M1g and M2g. Odds ratios were obtained from the training set.

* *P* < .05;

** *P* < .01;

*** *P* < .001.

### Explained variability and risk stratification through polygenic risk scores

Polygenic risk score contribution to the explained variance of models was relatively constant (NKR^2^ = 5.27%-6.12%; Table 3). When stratifying men into prostate cancer polygenic risk score quintiles, higher quintiles were associated with elevated odds ratios for MRI-positivity; increasing from an odds ratio of 0.56 (95% CI = 0.26 to 1.21) for the lowest polygenic risk score quintile (1%-20%) to 1.02 (95% CI = 0.49 to 2.13), 1.44 (95% CI = 0.69 to 2.98), and 2.72 (95% CI = 1.29 to 5.76) for the quintile of 21%-40%, 61%-80%, and 81%-100%, respectively. Similar observations were made using PI-RADS scores 1-3 vs 4-5 (Table S6).

**Table 3.** Diagnostic performance of individual decision strategies in predicting the magnetic resonance imaging outcome^a^

Model	Nongenetic models		Genetic models		
	AUC (95% CI)	NKR² (%)	AUC (95% CI)	NKR² (%)	Partial NKR^2^ (%)^b^
PI-RADS 1-2 vs 3-5					
M1	0.50 (0.37 to 0.63)	1.46	0.67 (0.55 to 0.79)	10.52	6.12
M2	0.51 (0.38 to 0.64)	1.89	0.67 (0.55 to 0.79)	10.76	5.97
PI-RADS 1-3 vs 4-5					
M1	0.62 (0.48 to 0.75)	4.05	0.80 (0.70 to 0.90)	12.23	5.69
M2	0.67 (0.53 to 0.79)	5.19	0.82 (0.72 to 0.90)	13.31	5.64

Abbreviations: AUC = area under the curve; CI = confidence interval; NKR^2^ = Nagelkerke R^2^; PI-RADS = Prostate Imaging–Reporting and Data System.

a Nongenetic models used age alone (M1) and in combination with serum prostate-specific antigen (M2). Both models had genetic versions denoted as M1g and M2g. AUC was reported for the holdout test set, and NKR^2^ was calculated in the training set.

b Contribution of the prostate cancer polygenic risk score compared with a base model including all predictors and covariates excluding prostate cancer polygenic risk score.

### Clinical utility of the prediction models

Nongenetic models M1 and M2 predicted MRI-positivity with an AUC of 0.50 (95% CI = 0.37 to 0.64) and 0.51 (95% CI = 0.38 to 0.64) in the holdout test set, respectively (Table 3). The inclusion of the polygenic risk scores in individual models resulted in improved performance, with an AUC of 0.67 (95% CI = 0.55 to 0.79; *P* = .026) and 0.67 (95% CI = 0.55 to 0.79; *P* = .039). Performance improved across all models when comparing PI-RADS scores 1-3 vs 4-5, with the best-performing model (M2g) using polygenic risk scores and PSA achieving an AUC of 0.82 (95% CI = 0.72 to 0.90; Table 3). Model performance was stable across men treated and untreated with 5α-reductase inhibitors and comparable with models calculated using the polygenic risk scores recently reported in the BARCODE1 study, adding to the robustness and reproducibility of our reported models (Tables S7 and S8). Calibration curves, sensitivity, and specificity for all models are reported in Figure S3 and Table S9. Details on the discrimination and calibration in the training set are shown in Table S10.

Decision curve analysis revealed that genetic models (M1g and M2g) provide greater clinical benefit than their nongenetic counterparts (M1 and M2) across all probabilities of interest (50%-100%) and MRI-risk groups (Figure 4). The use of the prostate cancer polygenic risk score for decision making substantially improved the net benefit of the nongenetic models, while models using age alone and combined with PSA (M1 and M2) performed inferiorly to the reference of recommending MRI in all men (treat all). Note that adding PSA to the genetic model did not improve the net benefit, as M1g and M2g performed almost identically. Models incorporating PSA density demonstrated further increases in net benefit (Figure S4).

**Figure 5.**
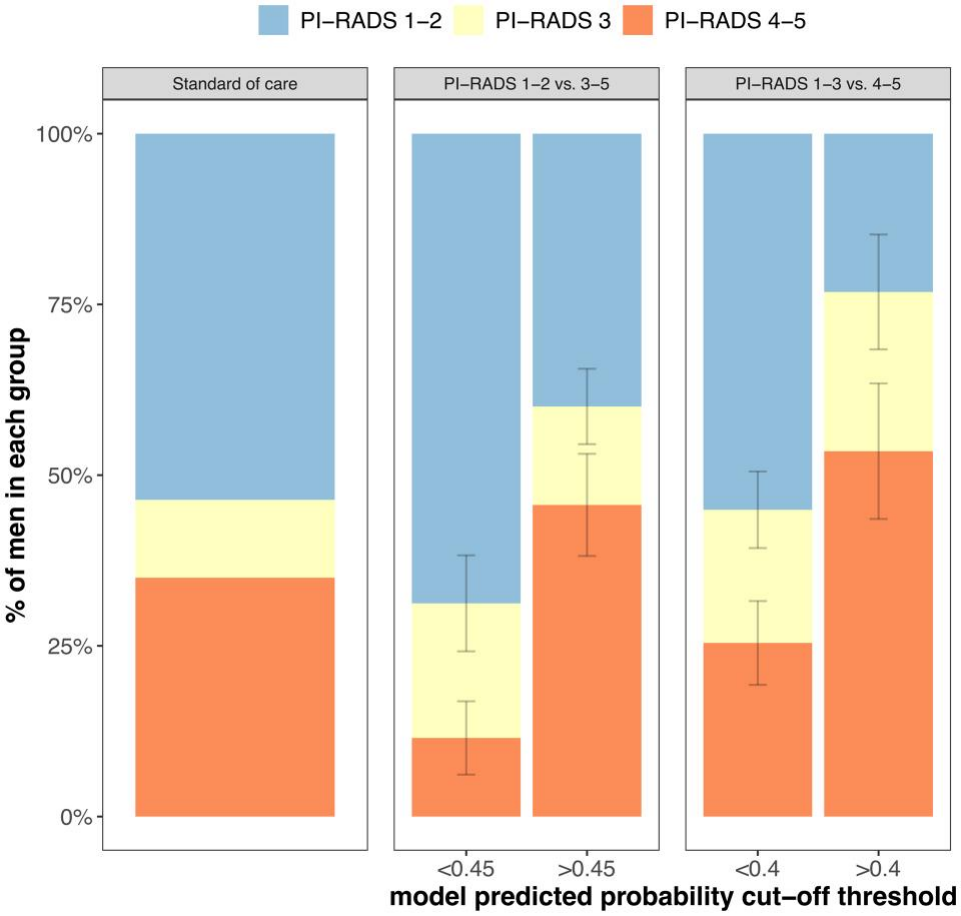
Proportions of MRI-positive men related to different pre-imaging risk stratification strategies in prostate cancer–suspected men. The diagram illustrates the distribution of PI-RADS scores in our cohort (standard of care) compared with scenarios where pre-MRI risk stratification is applied using the M1g model at thresholds of PI-RADS of at least 3 and PI-RADS of at least 4 defining MRI-positivity (middle and right columns, respectively). The probabilities of having a suspicious MRI finding were calculated in the holdout test sets (*n* = 77) using the respective training models. Overall, we chose a conservative cutoff for all decision strategies with a 0.45 cutoff for PI-RADS 1-2 vs 3-5 and 0.4 for PI-RADS 1-3 vs 4-5 genetic models, reducing the risk of missing men with cancer. The error bars represent the 95% confidence interval. Abbreviations: MRI = magnetic resonance imaging; PI-RADS = Prostate Imaging–Reporting and Data System.

Moreover, when considering the use of the genetic model M1g (age-based model) to manage men with clinically suspected cancer, the proportion of MRI-positive men increased from 46% using serum PSA levels alone to 60% (*P* = .11) (Figure 5). Aiming to stratify men with PI-RADS scores 1-3 vs 4-5 using the developed model led to a substantial increase of missed relevant findings (Figure 5). Notably, incorporating PSA density into the decision-making process increased the proportion of MRI-positive men to 67% (*P* = .03; Figure S5).

## Discussion

Efficient management of men with suspected prostate cancer is challenged by limited MRI capacity.^22^ Polygenic risk scores have shown a strong correlation with confirmed prostate cancer; however, their potential to improve pre-imaging risk stratification and increase the diagnostic yield of MRI has not been explored.^8^^,^^23-25^ Our study shows that polygenic risk scores associate with findings on prostate MRI (*P* < .001). Specifically, the highest clinical utility was demonstrated using a risk stratification model incorporating age and polygenic risk scores, increasing the proportion of men with suspicious findings at MRI (PI-RADS scores 3-5) compared with the use of PSA testing alone (*P* = .025).

The diagnostic accuracy and efficacy of the MRI pathway are limited by a high number of false-positive findings and negative MRI scans. Our consecutive prospective cohort included 46% MRI-negative men, with no difference in PSA values compared with MRI-positive men, which is consistent with guideline-defining studies such as the 4M trial.^4^ By incorporating polygenic risk score testing at initial assessment alongside PSA, our analysis indicates that clinicians could better identify men needing urgent MRI evaluation,^26^ which is consistent with findings from the Stockholm 3 trial^11^ and TARGET study.^27^ Adding genetic data to a PSA-gated screening strategy would enable more accurate triaging, with higher-risk patients receiving priority access to MRI, while lower-risk patients could be safely monitored. The demonstrated ability of polygenic risk scores to improve pre-MRI risk stratification could reduce unnecessary imaging costs while improving the identification of men who would benefit from immediate biopsy, thereby potentially improving clinically significant prostate cancer detection. The result would be a more efficient diagnostic process with shorter waiting times for those most likely to have aggressive cancers, addressing the need for more risk-adapted algorithms for early prostate cancer diagnosis.^28^^,^^29^ This improved efficiency could have downstream effects by reducing false-positives, limiting overdiagnosis of insignificant prostate cancer, and ultimately decreasing overtreatment, all of which carry considerable health-care costs. Further, polygenic risk scores are already gaining clinical recognition, as evidenced by the inclusion of polygenic risk score–based tests like Stockholm3 in the 2023 American Urological Association guidelines,^30^ and findings from the BARCODE1 study have further highlighted the potential impact of polygenic risk scores on improved and risk-adapted prostate cancer screening.^21^ In this context, we were able to demonstrate that by using a polygenic risk score derived from a more recent GWAS, the polygenic risk score–informed risk stratification was comparable with, and even slightly superior to, the polygenic risk scores used in the BARCODE1 study, adding to the robustness and generalizability of the proposed polygenic risk score–informed pre-MRI risk assessment.

Our study has some limitations. First, given the geographic region in which our study was conducted and aiming for robust polygenic risk score calculations in a rather small sample size, we included men of European ancestry only. Although we used transancestry GWAS summary statistics based on diverse ancestries,^8^ it is important to validate our results in diverse populations to avoid exacerbating health disparities.^31^ Second, the radiological reference standard might be limited because of interreader variability regarding thresholds for biopsy decisions.^32^ However, it can be assumed that the impact on differentiating MRI-negative and -positive men is minimal, as no statistically significant difference in diagnostic performance has been reported.^33^ Third, as recruitment was recently completed, histopathological results for MRI-positive findings and 3-year follow-up data^34^ were not yet available, limiting conclusions on overall clinical outcome. Fourth, genetically adjusted PSA values, family history, rare genetic mutations, and existing risk calculators were not considered in our analysis but could further contribute to a comprehensive genetics-informed risk-stratification approach.^35-37^ We also demonstrated that models incorporating PSA-density could further improve the diagnostic yield and overall net benefit among men undergoing MRI. However, because PSA-density in this analysis was MRI-derived and is not routinely available for pre-MRI risk stratification, these results are presented in the Supplementary materials only. Lastly, the generalizability of our results is limited by the single-center study design and model calibration using a rather small cohort, which calls for further investigation in a larger and more diverse cohort. Within these limitations we demonstrated the feasibility of using polygenic risk scores for pre-MRI risk stratification.

In conclusion, our findings underscore the concept and use of genotype-informed risk stratification to prioritize men for MRI who are likely to harbor clinically significant prostate cancer. Specifically, our concept could substantially increase the proportion of cancer-suspicious findings at MRI, while reliably identifying those who could safely avoid an immediate MRI. With further studies, polygenic risk scores could ultimately improve patient management by reducing resource inefficiencies.

## Supplementary Material

djag027_Supplementary_Data

## Data Availability

Because of the sensitive nature of patient data involved in this study and the absence of explicit consent from study participants, study data cannot be made publicly accessible. For any specific inquiries or collaboration requests, please contact the corresponding author.
