# Supplementary material for: Polygenic risk scores for premagnetic resonance imaging risk stratification in men with clinically suspected prostate cancer
Source: J Natl Cancer Inst. 2026 Feb 2;118(6):1063–72. doi: 10.1093/jnci/djag027 (PMC13247340; doi:10.1093/jnci/djag027)
Supplement: djag027_Supplementary_Data [file djag027_supplementary_data.pdf]

Supplementary Materials

Supplementary Methods

Electronic Data Capture System

For digital recording and organization of the data, an electronic data capture system was implemented in REDCap (1). This was intended to ensure that data were collected and validated in a standardized form. An electronic Case Report Form (eCRF) was created for each participant, in which all clinical data were centrally entered and documented. Data entry could occur in real-time via the data entry mask or subsequently by transferring from paper forms. In the latter case, data entry in the eCRF was to be checked by a second person to minimize transmission errors. New REDCap users received a brief introduction from the study staff at the beginning or had the opportunity to participate in a training course. Before the initial login to REDCap, appropriate user accounts had to be created by the administrator; for this, the study coordination could be contacted.

An annotated version of the ProGene codebook was automatically generated in REDCap and could be retrieved and exported at any time. The current version of the study protocol, study documents (information, informed consent, documentation forms, contact form), applicable SOPs, and ethics approvals were made available to all users in the REDCap data repository.

The software was provided by the Clinical Trial Office (CTO) of our institution. The general use of the software for clinical studies was approved by both the Ethics Committee and the Data Protection Office before its introduction. The database was routinely checked and secured through backups to prevent data loss or faulty data.

Collection and Documentation of Clinical Data

The initial documentation primarily included data on demographics, origin, pre-existing conditions and possible hospital stays, current medication, and familial prostate cancer risk. Patients were specifically asked about the intake of 5-alpha-reductase inhibitors and any concomitant medication. The corresponding pharmacological agents were subsequently coded based on the Anatomical Therapeutic Chemical (ATC) classification. Pre-existing conditions and diagnoses made during hospital stays were classified according to ICD-10. Furthermore, suspicious DRE and/or TRUS findings were queried or documented based on letters from urological practices. For traceability, medical reports and other

documents about prior medical examinations were inserted in anonymized form at the appropriate place in the electronic data capture system. Prostate-specific antigen (PSA) values were queried or extracted from their medical record. For traceability, laboratory findings were anonymized and entered at the appropriate place in the eCRF.

Genetic Sampling and Sample Management

Sample Collection and Storage

To collect saliva samples, OraGene OG-510 (Genotek, Ottawa, Ontario, Canada) saliva kits were used. This non-invasive method was suitable for obtaining biological samples, particularly for genetic studies. Medical expertise was not required; collection followed the manufacturer's official instructions. To maintain privacy, all saliva tubes were labeled with the assigned study ID.

The sample was stored at room temperature, between 15 and 30 °C, in a standard sample box. Finally, the sample was inventoried under the assigned study ID in designated sample lists. The sample boxes were stored in lockable cabinets at the respective recruitment center, accessible only to study staff.

DNA Extraction

The collected saliva samples were pre-sorted in batches (sample number was either a multiple of 24 or 96, minimum ≥96 samples) for genetic material isolation. The DNA extraction was commissioned by the Laboratory for Statistical Genetics to an external service provider and followed an established extraction protocol. After DNA extraction, UV-Vis spectrophotometry was performed on all purified samples, using absorption spectrum ratios (A260/A280 and A260/A230) to assess DNA quality. Additionally, an agarose gel electrophoresis was conducted on a randomly selected sample (10–20 samples per batch) to evaluate DNA integrity. Because UV-Vis measurements were more susceptible to contaminants and tended to overestimate DNA concentration, a fluorescence-based DNA quantification was performed to selectively measure double-stranded DNA (PicoGreen™, Thermo Fisher Scientific, PA, USA). Aliquots of the DNA stock samples were standardized to a concentration of 50 ng/µl and a total volume of 4 µl for genotyping preparation.

External Service Provider for DNA Extraction: LGC Genomics GmbH (Berlin, Germany)

Genotyping

For characterizing common genetic variants, genome-wide SNP arrays were used to systematically capture genome variability. All DNA samples from this study were genotyped using the Infinium® Global Screening Array-24 v3.0 (GSA) BeadChip (Illumina, San Diego, CA, USA). The GSA was a state-of-the-art chip for parallel genotyping of over 700,000 SNPs, selected especially for their clinical relevance. Genotyping was externally performed by the Human Genotyping Facility (HuGe-F) at Erasmus Medical Center.

Institution Commissioned with Genotyping: Erasmus Medical Center, Human Genotyping Facility (HuGe-F; Rotterdam, Netherlands)

Biobanking

After initial genotyping, all DNA stock samples and, if applicable, additional normalized aliquots were transferred to the local biobank at our institution. The genetic material was securely stored in 96-well plates at –60 °C. This ensured long-term storage of the biological samples, allowing future genetic studies without requiring new samples from participants.

Quality control

Genetic quality control was performed in Ricopili using these default settings:

- autosomal heterozygosity deviation (Fhet<0.02)
- relatedness (identity by descent segments [PI-HAT >0.2]; retaining one in pair, preferring cases)
- SNP missingness (<0.05)
- subject missingness (<0.02)
- minor allele frequency (>0.01)
- Hardy-Weinberg disequilibrium (controls: p>10e-6, cases: p>10e-10)

References:

1. Harris, P. A., Taylor, R., Thielke, R., Payne, J., Gonzalez, N., & Conde, J. G. Research electronic data capture (REDCap)—a metadata-driven methodology and workflow process for providing translational research informatics support. Journal of biomedical informatics 2009, 42 (2), 377-381.

Supplementary Results

Association of individual PSA-density with MRI findings

PSA-density was the only non-genetic marker demonstrating a significant difference between MRI-negative and -positive men with an OR 4.94 (95%CI: 2.85-8.58; p<.001) (Table S4); the OR increased to 5.07 (95%CI: 2.98-8.64; p<.001) looking at men with PI-RADS scores 1-3 vs. 4-5. When incorporated into prediction models, PSA-density was a statistically significant predictor in all models (M3g; **Table S5** and **Figure S4**).

Clinical utility of the prediction models incorporating PSA-density

The non-genetic model M3 predicted MRI-positivity with an AUC of 0.71 (95%CI: 0.59-0.83) in the hold-out test set (**Table S8)**. The inclusion of the PRS in this model resulted in improved performance, with an AUC of 0.75 (95%CI: 0.63-0.86; p=.29). Performance improved across all models when comparing PI-RADS scores 1-3 vs. 4-5, with the best-performing model (M3g) using PRS and PSA-density achieving an AUC of 0.88 (95%CI: 0.80-0.95).

The use of PSA-density for decision-making substantially improved the net benefit of the non-genetic models. Overall, the PSA-density-based genetic model (M3g) demonstrated the highest clinical utility across all thresholds (**Figure S4**). Moreover, when considering the use of genetic models M1g or M3g (age- and PSA-density-based models) to manage men with clinically suspected cancer, the proportion of MRI-positive men increased from 46% using serum PSA levels alone to 67% (p=.025) (**Figure S5A**). In comparison, using a PSA-density cut-off of 0.1 for decision-making increased the proportion of MRI-positive men by 8% (46% vs. 54%; p=.24), while the risk of missing men with relevant findings (PI-RADS 4-5) was 15%. Aiming to stratify men with PI-RADS scores 1-3 vs. 4-5 using the developed models led to a substantial increase of missed relevant findings (**Figure S5B**).

Supplementary Tables

Table S1. Logistic regression models trained.

Non-Genetic models			Genetic models*	
Model	Name	Independent variables	Name	Independent variables
M1	M1: Demographic	Age	M1g: Genetic	Age + PRS + PC 1-4
M2	M2: Clinical	Age + PSA	M2g: Genetic + clinical	Age + PRS + PC 1-4 + PSA
M3	M3: Density	Age + PSA-D	M3g: Genetic + density	Age + PRS + PC 1-4 + PSA-D

* Genetic equivalent of non-genetic model. PC = principal component; PRS= polygenic risk score; PSA= prostate-specific antigen; PSA-D= prostate-specific antigen density

Table S2. Sample characteristics of PI-RADS cut-off 1-3 vs. 4-5

	PI-RADS 1-3 vs. 4-5 outcome	
**Characteristic**	**PI-RADS 1-3**<br>N= 251	**PI-RADS 4-5**<br>N= 135
**Age (years)**	64 (59, 70)	69 (62, 74)
**BMI (kg/m²)**	25.9 (24.3, 28.4)	25.8 (24.0, 29.3)
Missing	10	6
**Previous biopsy**		
No previous biopsy	179 (76%)	115 (88%)
Previous biopsy & no malignancy	58 (24%)	16 (12%)
Missing	14	4
**Intake of 5 α-Reductase-Inhibitors**	18 (7%)	5 (4%)
Missing	1	0
**Serum PSA (ng/ml)**	5.9 (4.6, 8.0)	6.3 (5.1, 9.1)
**Prostate volume (ml)**	60 (43, 73)	40 (30, 52)
**PSA-density (ng/mL/cc)**	0.10 (0.08, 0.14)	0.16 (0.12, 0.22)
**Scaled PRS**	-0.25 (-0.90, 0.49)	0.31 (-0.38, 0.97)
**PI-RADS version**		
2	95 (38%)	67 (50%)
2.1	156 (62%)	68 (50%)
**PI-RADS**		
1	6 (2%)	-
2	201 (80%)	-
3	44 (18%)	-
4	-	79 (59%)
5	-	56 (41%)

Note.- Continuous variables are reported as median and (interquartile range); categorical variables are reported as number and %. P-values were calculated with Fisher's exact test. BMI= body mass index; N= number; PI-RADS= Prostate Imaging–Reporting and Data System; PSA= prostate-specific antigen

Table S3. Sample characteristics of the test and train set.

Characteristic	Test set, N = 77	Train set, N = 309	p-value
**Age (years)**	65 (58, 72)	65 (60, 72)	0.8
**Previous biopsy**			0.037
No previous biopsy	55 (71%)	239 (82%)	
Previous biopsy & no malignancy	22 (29%)	52 (18%)	
(Missing)	0	18	
**Intake of 5 α-Reductase-Inhibitors**	3 (4%)	20 (6%)	0.6
(Missing)	0	1	
**Serum PSA (ng/ml)**	6.7 (5.1, 8.9)	6.0 (4.7, 8.0)	0.063
**Prostate volume (ml)**	57 (40, 73)	51 (36, 67)	0.2
**PSA-density (ng/mL/cc)**	0.12 (0.08, 0.17)	0.12 (0.08, 0.17)	0.9
**PI-RADS version**			<0.001
2	0 (0%)	162 (52%)	
2.1	77 (100%)	147 (48%)	
**PI-RADS**			0.3
1	2 (3%)	4 (1%)	
2	39 (51%)	162 (52%)	
3	13 (17%)	31 (10%)	
4	15 (19%)	64 (21%)	
5	8 (10%)	48 (16%)	
**MRI Outcome**			>0.9
PI-RADS 1-2	41 (53%)	166 (54%)	
PI-RADS 3-5	36 (47%)	143 (46%)	

Note.- Continuous variables are reported as median and (interquartile range); categorical variables are reported as number and %. Tests for significance used where Wilcoxon rank sum test, Pearson's Chi-sqaured test and Fischer's exact test. BMI= body mass index; N= number; PCa = Prostate cancer; PI-RADS= Prostate Imaging–Reporting and Data System; PSA= prostate-specific antigen

Table S4. Logistic regression of unadjusted independent markers.

	OR (95% CI)	p-value
PRS	1.56 (1.23-1.98)	<0.001
PSA	1.17 (0.93-1.46)	0.18
PSA-D	4.94 (2.85-8.58)	<0.001
PC1	1.19 (0.95-1.5)	0.12
PC2	0.79 (0.63-0.99)	0.04
PC3	0.99 (0.79-1.24)	0.93
PC4	0.98 (0.79-1.23)	0.89
Age	1.24 (0.99-1.55)	0.07

CI= confidence interval; PC= principal component; PSA= serum prostate-specific antigen; PSA-D= prostate-specific antigen density; PRS= polygenic risk score

Table S5. Association of independent markers with positive MRI findings, extended.

	PI-RADS 1-2 vs. 3-5 outcome						PI-RADS 1-3 vs. 4-5 outcome					
	Non-genetic models			Genetic models			Non-genetic models			Genetic models		
	M1	M2	M3	M1g	M2g	M3g	M1	M2	M3	M1g	M2g	M3g
Marker	OR (95% CI)	OR (95% CI)	OR (95% CI)	OR (95% CI)	OR (95% CI)	OR (95% CI)	OR (95% CI)	OR (95% CI)	OR (95% CI)	OR (95% CI)	OR (95% CI)	OR (95% CI)
Age	1.24 (0.99-1.56)	1.21 (0.96-1.53)	1.28 (1.00-1.65)	1.30 * (1.02-1.65)	1.28 * (1.00-1.63)	1.33 * (1.03-1.74)	1.45 ** (1.14-1.86)	1.40 ** (1.09-1.80)	1.60 *** (1.22-2.13)	1.52 ** (1.18-1.98)	1.47 ** (1.14-1.92)	1.68 *** (1.27-2.26)
PSA		1.12 (0.89-1.42)			1.10 (0.86-1.40)			1.22 (0.96-1.55)			1.23 (0.96-1.59)	
PSA-density			4.94 *** (2.95-8.80)			4.50 *** (2.68-8.05)			5.45 *** (3.28-9.59)			5.30 *** (3.15-9.49)
PRS				1.58 *** (1.24-2.04)	1.58 *** (1.24-2.03)	1.57 ** (1.21-2.06)				1.59 *** (1.23-2.07)	1.59 *** (1.23-2.08)	1.65 ** (1.24-2.23)

Note. – Non-genetic models used age alone (M1) and in combination with serum PSA (M2) and PSA-D (M3). All three models had genetic versions denoted as M1g, M2g, and M3g. ORs were obtained from the training set. OR= odds ratio; CI= confidence interval; PSA= serum prostate-specific antigen; PRS= polygenic risk score for PCa; *= p<.05; **= p<.01; ***= p<.001

Table S6. Odds Ratios of stratified PRS in both outcomes

	PI-RADS 1-2 vs. 3-5			PI-RADS 1-3 vs. 4-5		
PRS quintile	number in each quintile	OR (95% CI)	% of PI-RADS 3-5 in quintile	number in each quintile	OR (95% CI)	% of PI-RADS 4-5 in quintile
0-20%	62	0.56 (0.26-1.21)	29.0	62	0.71 (0.3-1.66)	21.0
20-40%	62	1.02 (0.49-2.13)	43.5	62	1.32 (0.6-2.91)	33.9
40-60%	62	1.00 (ref.)	43.5	62	1.00 (ref.)	29.0
60-80%	62	1.44 (0.69-2.98)	50.0	62	1.77 (0.81-3.87)	38.7
80-100%	61	2.72 (1.29-5.76)	65.6	61	3.16 (1.45-6.89)	52.5

Note. – Squared brackets include stated number and round brackets do not include stated number.
OR= odds ratio; CI= confidence interval; PRS= polygenic risk score

Table S7. Sensitivity analysis of cohort without 5α-reductase inhibitors

Model	Models developed without 5α-reductase inhibitors AUC (95% CI)	Original models AUC (95% CI)
M1	0.51 (0.37–0.64)	0.50 (0.37–0.63)
M2	0.50 (0.37–0.64)	0.51 (0.38–0.64)
M3	0.68 (0.54–0.80)	0.71 (0.60–0.83)
M1g	0.69 (0.56–0.81)	0.67 (0.55–0.79)
M2g	0.68 (0.55–0.80)	0.67 (0.55–0.79)
M3g	0.74 (0.62–0.85)	0.75 (0.63–0.86)

Non-genetic models used age alone (M1) and in combination with serum PSA (M2) and PSA-D (M3). All three models had genetic versions denoted as M1g, M2g, and M3g. AUC= area under the curve; CI= confidence interval; PRS= polygenic risk score

Table S8. Diagnostic performance of different PRS.

	PRS used by the BARCODE 1 study			PRS by Conti et al.		
Model	AUC (95%CI)	NKR² (%)	Partial NKR2 (%)*	AUC (95%CI)	NKR² (%)	Partial NKR2 (%)*
M1g	0.59 (0.47-0.71)	8.93	4.45	0.67 (0.55-0.79)	10.52	6.12
M2g	0.60 (0.48-0.73)	9.23	4.35	0.67 (0.55-0.79)	10.76	5.97
M3g	0.74 (0.63-0.85)	23.56	2.43	0.75 (0.63-0.86)	25.78	5.27

Note. – Each model incorporated PRS with the first principal components along with age (M1g), in combination with serum PSA (M2g) and PSA-density (M3g). AUC was reported for the hold-out test set while NKR2 was calculated in the train set. *Contribution of the PCa-PRS compared to a base model including all predictors and covariates excluding PCa-PRS. AUC= Area under the curve; CI= confidence interval; NKR2= Nagelkerke's R2; PSA= serum prostate-specific antigen; PRS= polygenic risk score

Table S9. Ideal sensitivity, specificity, specificity at 91% sensitivity (95% CI), in hold-out test set.

	PI-RADS cut-off 1-2 vs. 3-5						PI-RADS cut-off 1-3 vs. 4-5					
	Non-Genetic Models			PRS			Non-Genetic Models			PRS		
	Ideal			Ideal			Ideal			Ideal		
	Sensitivity (95% CI)	Specificity (95% CI)	Specificity (95% CI) at 91% Sensitivity	Sensitivity (95% CI)	Specificity (95% CI)	Specificity (95% CI) at 91% Sensitivity	Sensitivity (95% CI)	Specificity (95% CI)	Specificity (95% CI) at 91% Sensitivity	Sensitivity (95% CI)	Specificity (95% CI)	Specificity (95% CI) at 91% Sensitivity
M1	0.333 (0.056-0.472)	0.78 (0.415-0.902)	0 (0-0.335)	-	-	-	0.481 (0.186-0.667)	0.76 (0.369-0.898)	0.237 (0.02-0.426)	-	-	-
M2	0.472 (0.194-0.639)	0.659 (0.268-0.805)	0.146 (0-0.317)	-	-	-	0.667 (0.346-0.818)	0.7 (0.239-0.857)	0.18 (0.083-0.426)	-	-	-
M3	0.583 (0.361-0.723)	0.854 (0.512-0.976)	0.073 (0-0.537)	-	-	-	0.704 (0.424-0.875)	0.84 (0.522-0.979)	0.16 (0.02-0.776)	-	-	-
M1g	-	-	-	0.694 (0.36-0.861)	0.659 (0.39-0.78)	0.098 (0.024-0.537)	-	-	-	0.704 (0.219-0.846)	0.82 (0.545-0.918)	0.54 (0.128-0.746)
M2g	-	-	-	0.722 (0.333-0.861)	0.659 (0.293-0.78)	0.122 (0.024-0.537)	-	-	-	0.704 (0.286-0.864)	0.8 (0.527-0.92)	0.56 (0.319-0.739)
M3g	-	-	-	0.639 (0.361-0.778)	0.878 (0.39-0.976)	0.195 (0.073-0.488)	-	-	-	0.852 (0.524-0.964)	0.82 (0.377-0.92)	0.7 (0.185-0.898)

Note. – Non-genetic models used age alone (M1) and in combination with serum PSA (M2) and PSA-density (M3). All three models had genetic versions denoted as M1g, M2g, and M3g. CI = confidence interval; PSA= serum prostate-specific antigen; PRS= polygenic risk score

Table S10. Discrimination and Calibration, in train set.

	PI-RADS cut-off 1-2 vs. 3-5						PI-RADS cut-off 1-3 vs. 4-5					
	Discrimination (AUC)		Calibration				Discrimination (AUC)		Calibration			
	Non-Genetic Models	Genetic Models	Non-Genetic Models		Genetic Models		Non-Genetic Models	Genetic Models	Non-Genetic Models		Genetic Models	
			Intercept	Slope	Intercept	Slope			Intercept	Slope	Intercept	Slope
M1	0.57	-	0.11	1317	-	-	0.607	-	0.136	1358	-	-
M2	0.549	-	0.15	1328	-	-	0.601	-	-0.014	1072	-	-
M3	0.713	-	-0.01	0.997	-	-	0.756	-	0.107	1126	-	-
M1g	-	0.632	-	-	-0.068	0.791	-	0.639	-	-	-0.197	0.664
M2g	-	0.63	-	-	-0.067	0.747	-	0.647	-	-	-0.186	0.687
M3g	-	0.737	-	-	-0.047	0.925	-	0.762	-	-	-0.058	0.873

Note. – Non-genetic models used age alone (M1) and in combination with serum PSA (M2) and PSA-density (M3). All three models had genetic versions denoted as M1g, M2g, and M3g.CI = confidence interval; PSA= serum prostate-specific antigen; PRS = polygenic risk score

Supplementary Figures

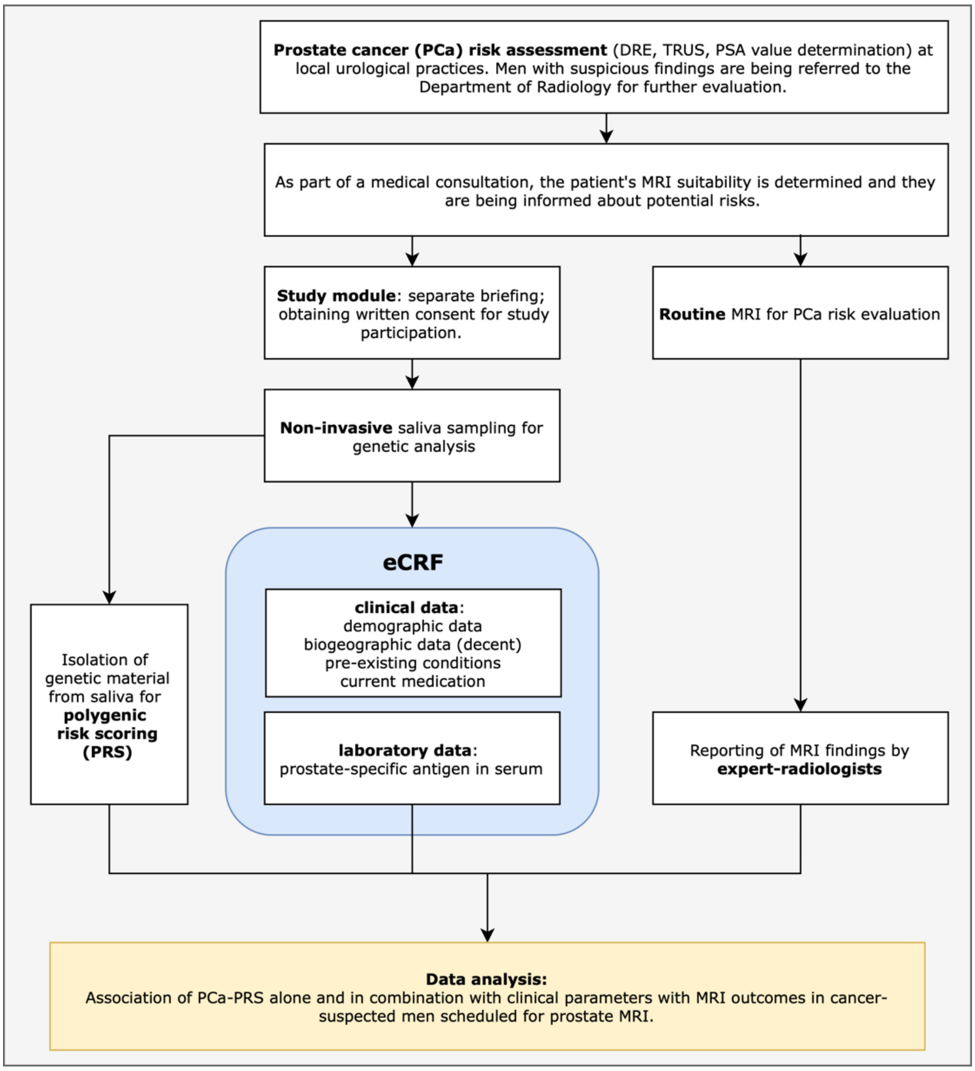

Figure S1: Data collection and study workflow.

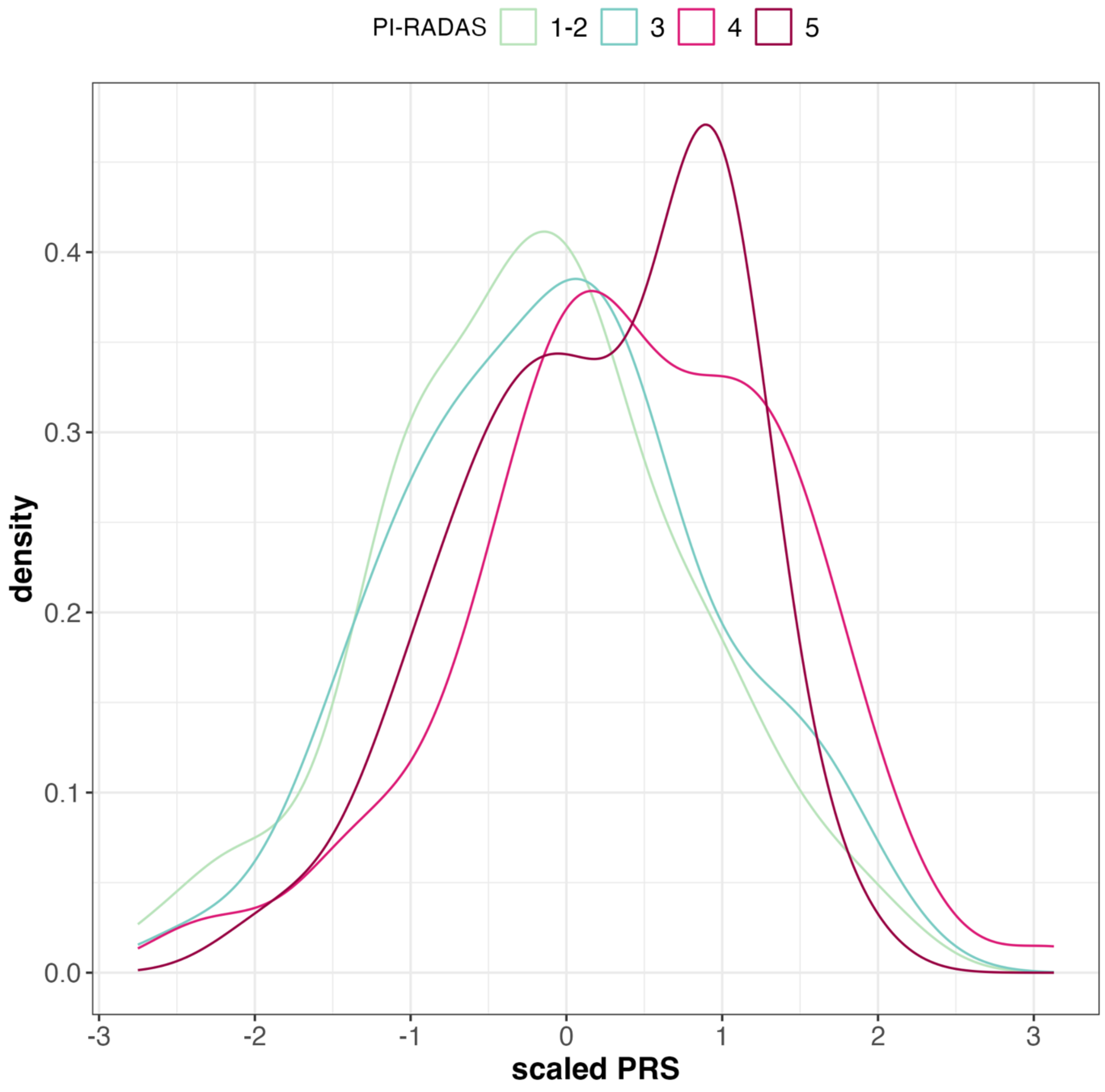

Figure S2: Polygenic risk score density curves separated by PI-RADS score.

Prostate Imaging–Reporting and Data System (PI-RADS) score 1 was grouped with PI-RADS score 2 as this score only accounts for six patients in our sample. PRS= polygenic risk score

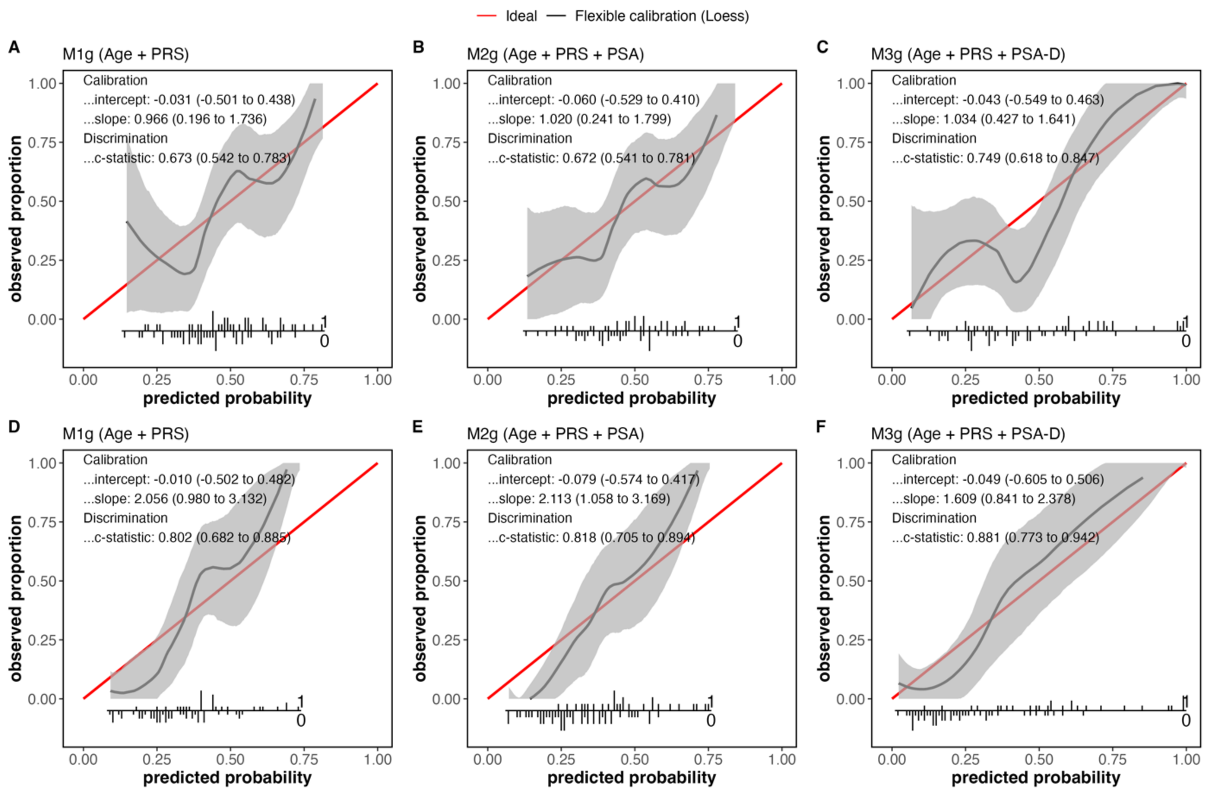

Figure S3: Calibration curves for the developed genetic models using a PI-RADS cut-off 1-2 vs. 3-5 (A-C) and 1-3 vs. 4-5 (D-F).

Calibration assessed in test set. Plots show how predicted probabilities align with true observed proportion. The red line is the ideal and represents a perfectly calibrated model. The black line is the respective model with a loses function applied to it. Grey area represents the 95% CI. Caution needs to be undertaken when interpreting these plots, as our test set size is quite small (N=77) and does not meet recommendations. PSA= prostate specific antigen; PSA-D= prostate specific antigen density; PRS= polygenic risk score

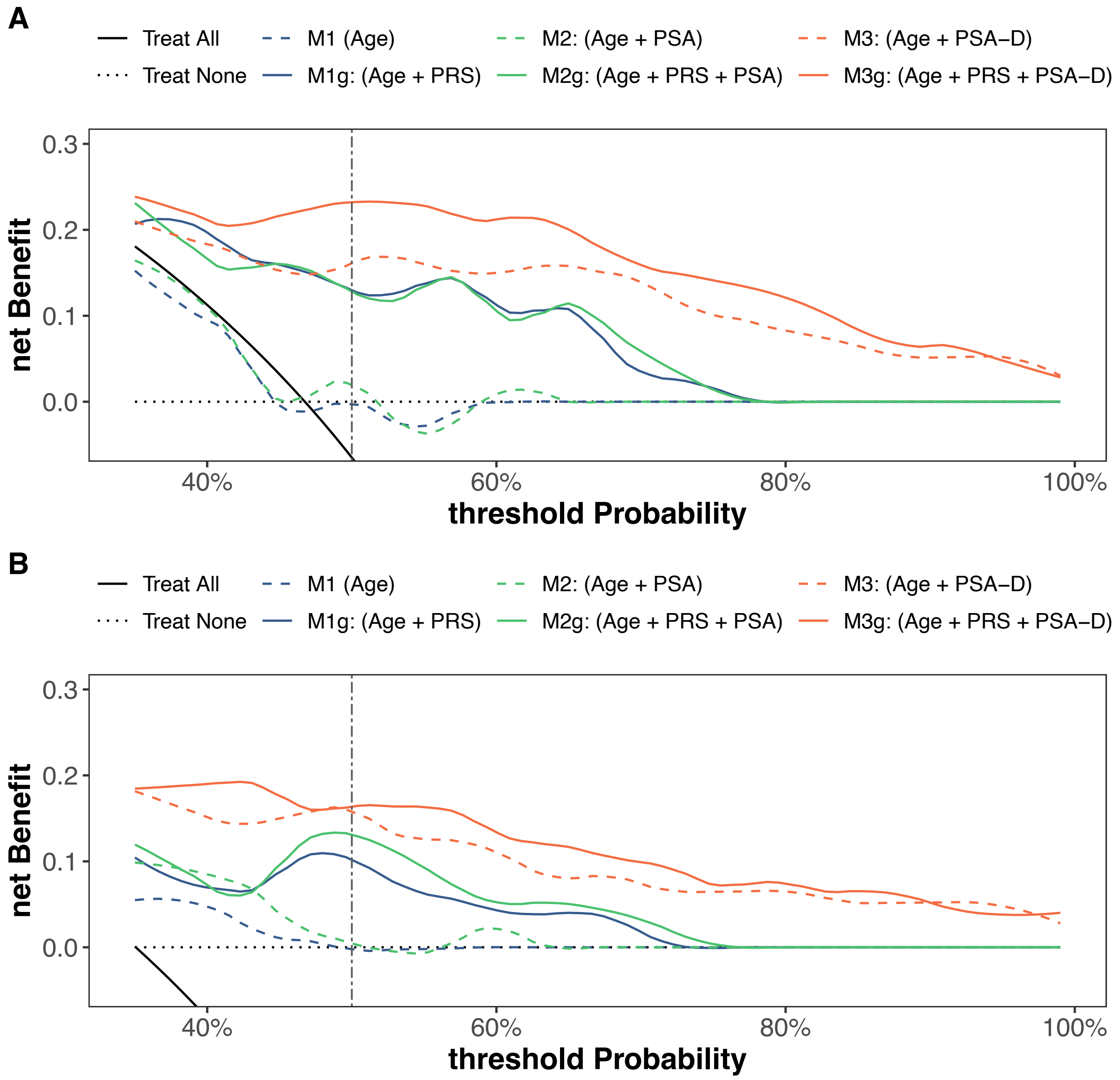

Figure S4: Decision curve analysis comparing clinical utility of different decision strategies for pre-MRI decision making men with suspected prostate cancer in comparison to PSA-density models.

Decision curve analysis was performed in the hold-out test set of 77 men **A** for the outcome of PI-RADS scores 1-2 vs. 3-5 and **B** for PI-RADS scores 1-3 vs. 4-5. Decision curve analysis is used to evaluate prediction models, by comparing their net benefit. It simulates two scenarios: in one all the men would undergo imaging (treat all) and in the other none would (treat none). Clinically useful decision strategies lie above these scenarios. The graph gives the expected net benefit per participant relative to image none. At a 50% threshold for MRI (every other MRI scan shows PI-RADS score 3-5 lesions), all genetic models had a higher net benefit compared to their non-genetic counterparts. Note how the genetic model using PSA-density (M3g) outperforms all other strategies at any given threshold. PI-RADS=

Prostate Imaging–Reporting and Data System; PSA: prostate-specific antigen; PSA-D: prostate-specific antigen density; PRS=polygenic risk score

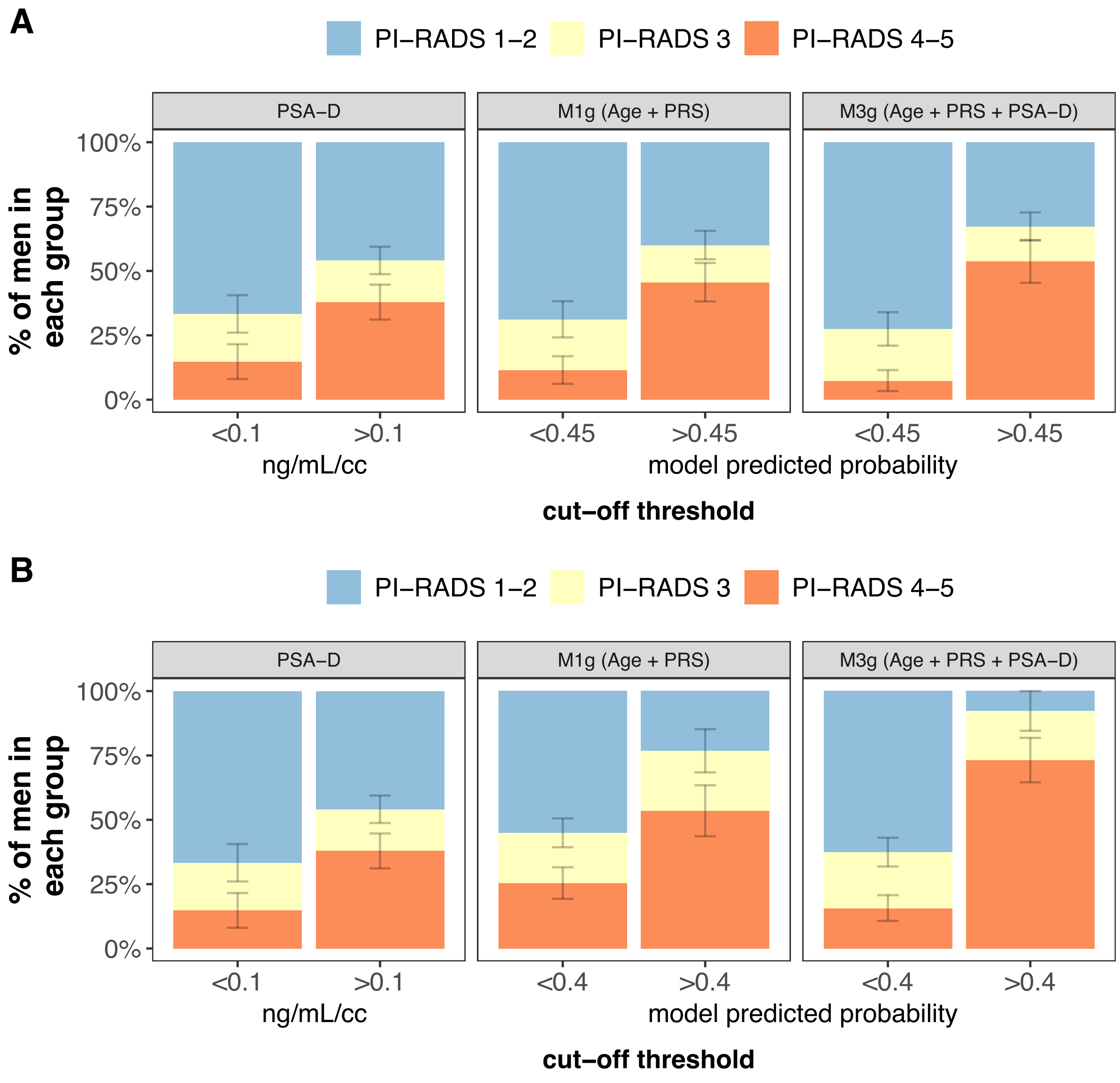

Figure S5: Proportions of MRI-positive men related to different pre-imaging risk stratification strategies in prostate cancer suspected men.

A Proportions of men with negative (PI-RADS 1-2), equivocal (PI-RADS 3) and suspicious (PI-RADS 4-5) MRI findings, related to different decision strategies using PSA-density only and genetic models using age alone or in combination with PSA-density (M1g and M3g, respectively). The probability of having a suspicious MRI finding (PI-RADS 1-2 vs. 3-5) was calculated in the hold-out test sets (n=77) using the respective training models. Overall, we chose a conservative cut-off for all decision strategies with a cut-off for PSA-density of 0.1 ng/mL/cc and 0.45 for genetic models, reducing the risk of missing men with cancer. The diagram illustrates that MRI-negative men (PI-RADS score 1-2) are more likely to have a genetic risk threshold below 0.45 than a PSA-density below 0.1. The error bars represent the 95% confidence interval. **B** shows the same diagram, but results gathered from the models with the PI-

RADS outcome of 1-3 vs. 4-5. Here we choose a threshold of 0.4 as this resulted in higher sensitivities. When using M3g only 8% had unsuspicious MRI results (PI-RADS 1-2) among those that showed a higher predictive probability of suspicious MRI lesions (>0.4), this is the lowest number of false negatives of any of our decision thresholds. PSA-D: prostate-specific antigen density; PI-RADS= Prostate Imaging–Reporting and Data System
